# From Dental Procedures to Endocarditis: Cardiobacterium hominis Infection of a Bioprosthetic Mitral Valve

**DOI:** 10.7759/cureus.85411

**Published:** 2025-06-05

**Authors:** Ashley Sundin, Paul Duesing, Ashwin Jagadish, Mohannad Al Akeel, Venkata Vedantam, Neethu Vedantam, Mathew Finniss

**Affiliations:** 1 Internal Medicine, James H. Quillen College of Medicine, East Tennessee State University, Johnson City, USA; 2 Infectious Diseases, James H. Quillen College of Medicine, East Tennessee State University, Johnson City, USA

**Keywords:** bioprosthetic mitral valve, cardiobacterium hominis, endocarditis, infective endocarditis, prosthetic valve endocarditis

## Abstract

Prosthetic valve endocarditis (PVE) is an extremely rare but serious complication in patients with bioprosthetic heart valves following procedures such as dental work, especially when prophylactic antibiotics are not administered. We present the case of a 67-year-old male with a bioprosthetic mitral heart valve who developed subacute endocarditis caused by *Cardiobacterium hominis *after undergoing a dental procedure without antibiotic prophylaxis. The patient presented to the emergency department with a six-month history of worsening fatigue, weakness, intermittent fevers, and lower extremity edema. Initial evaluation with a transthoracic echocardiogram suggested infective endocarditis. A follow-up transesophageal echocardiogram confirmed bioprosthetic mitral valve endocarditis with large vegetations and paravalvular regurgitation. While admitted, blood cultures obtained from the patient's primary care physician did come back positive for *C. hominis,* confirming the diagnosis of *C. hominis* subacute endocarditis. During the patient's hospitalization, the patient developed multiple sequelae, including septic emboli to the spleen and significant anemia due to macroangiopathic hemolysis. The patient was discharged after initiation of intravenous (IV) ceftriaxone and underwent redo mitral valve replacement. He had a successful postoperative recovery and continued IV ceftriaxone for six weeks. This case highlights the diagnostic challenges of rare pathogens in PVE, particularly *C. hominis*, and emphasizes the importance of timely diagnosis, appropriate antibiotic therapy, and prophylactic antibiotics for high-risk patients undergoing invasive procedures.

## Introduction

Prosthetic valve endocarditis (PVE) is a rare and life-threatening type of infective endocarditis that is a sequel of bioprosthetic cardiac valve replacements. PVE has a high morbidity and mortality and is the most severe form of infective endocarditis [1]. The mortality rate can range from 26% to 5% in those who are treated medically and 23% to 43% in those who are treated surgically [2]. It often arises after invasive procedures, such as dental work, particularly in the absence of appropriate antibiotic prophylaxis. Most cases of PVE are caused by more common pathogens, such as *Streptococcus*, *Staphylococcus*, and *Enterococcus* species. Endocarditis caused by HACEK (*Haemophilus*, *Actinobacillus*, *Cardiobacterium*, *Eikenella*, and *Kingella*) organisms accounts for only 1%-6% of infective endocarditis cases, and among the HACEK organisms, *Cardiobacterium hominis *is an exceptionally rare cause of infective endocarditis [3-5]. With only 44 cases of *C. hominis* infective endocarditis being reported in the literature from 2000-2022 and only 15 cases of *C. hominis* PVE reported until 2006, we present the rare case of a patient diagnosed with subacute *C. hominis* PVE after mitral valve replacement [6,7]. We also highlight the diagnostic and treatment challenges associated with such a novel disease process [6].

This case aims to add to the growing body of medical knowledge on the diagnostic and therapeutic challenges posed by rare pathogens like *C. hominis* in PVE. We also want to emphasize the critical importance of antibiotic prophylaxis for patients with bioprosthetic valves, particularly in the context of dental procedures, to prevent such rare but serious infections [4]. Preventing such a serious form of endocarditis will preserve patients’ lives and decrease possible sequelae from this disease process. This case report underscores the need for early detection and tailored management to improve patient outcomes in cases of infective endocarditis caused by *C. hominis*.

## Case presentation

A 67-year-old male with history of mitral valve regurgitation due to Barlow's mitral valve disease, a condition involving the mitral valve leaflets entering the left atrium during contraction, bi-leaflet scallop prolapse status post recent bioprosthetic valve replacement, maze procedure for atrial fibrillation approximately one year ago, melanoma, chronic normocytic anemia, and atrial fibrillation who was sent to the emergency department due to positive blood cultures obtained from his primary care physician. The patient had an extensive outpatient workup for continued weakness and generalized fatigue with lower extremity swelling that eventually led to outpatient blood cultures being obtained. The patient had been struggling with a six-month history of increasing weakness, fatigue, chills, lower extremity edema, and several recent intermittent fevers with a maximum temperature of 103 ˚F. The patient noted that he had had a root canal and multiple reparative dental procedures without dental implants done several months earlier, without prophylactic antibiotics.

**Figure 1:**
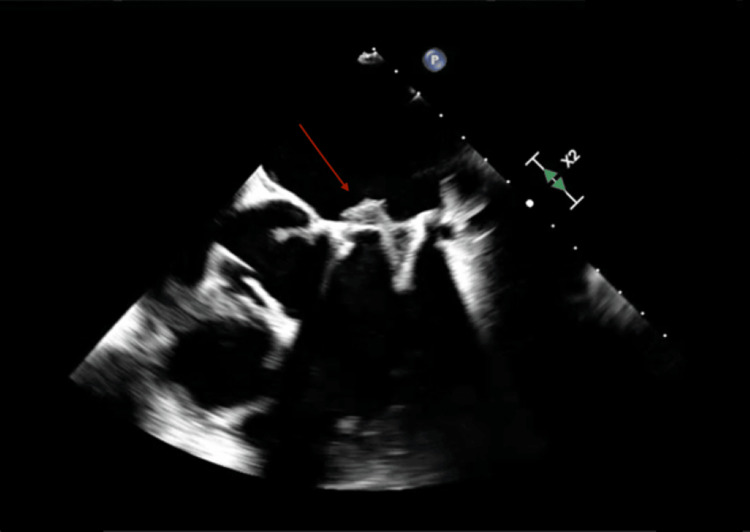
Transesophageal echocardiography with evidence of large vegetation on the anterior right cusp indicated by the red arrow.

On admission, the patient’s blood pressure was 118/67 mmHg, heart rate 78 beats per minute, respiratory rate was 16 breaths per minute, oxygen saturation was 100% on room air, and temperature was 98.0 ˚F. A transthoracic echocardiogram (TTE) showed a bioprosthetic mitral valve with vegetations, significant valve degeneration, mild-to-moderate mitral valve regurgitation, and an elevated gradient pressure of 10-11 mmHg, suspicious for endocarditis. Infectious disease was consulted and recommended a transesophageal echocardiogram (TEE), and antibiotics that included intravenous cefepime and vancomycin. Cardiology was consulted for subacute endocarditis, with echocardiography showing significant prosthetic valve dysfunction/vegetation. The TEE ordered and confirmed bioprosthetic mitral valve endocarditis with a large vegetation on the anterior right cusp with a moderate degree of stenosis as well as paravalvular regurgitation with no significant prosthesis leaking (Figure 1).

**Figure 2:**
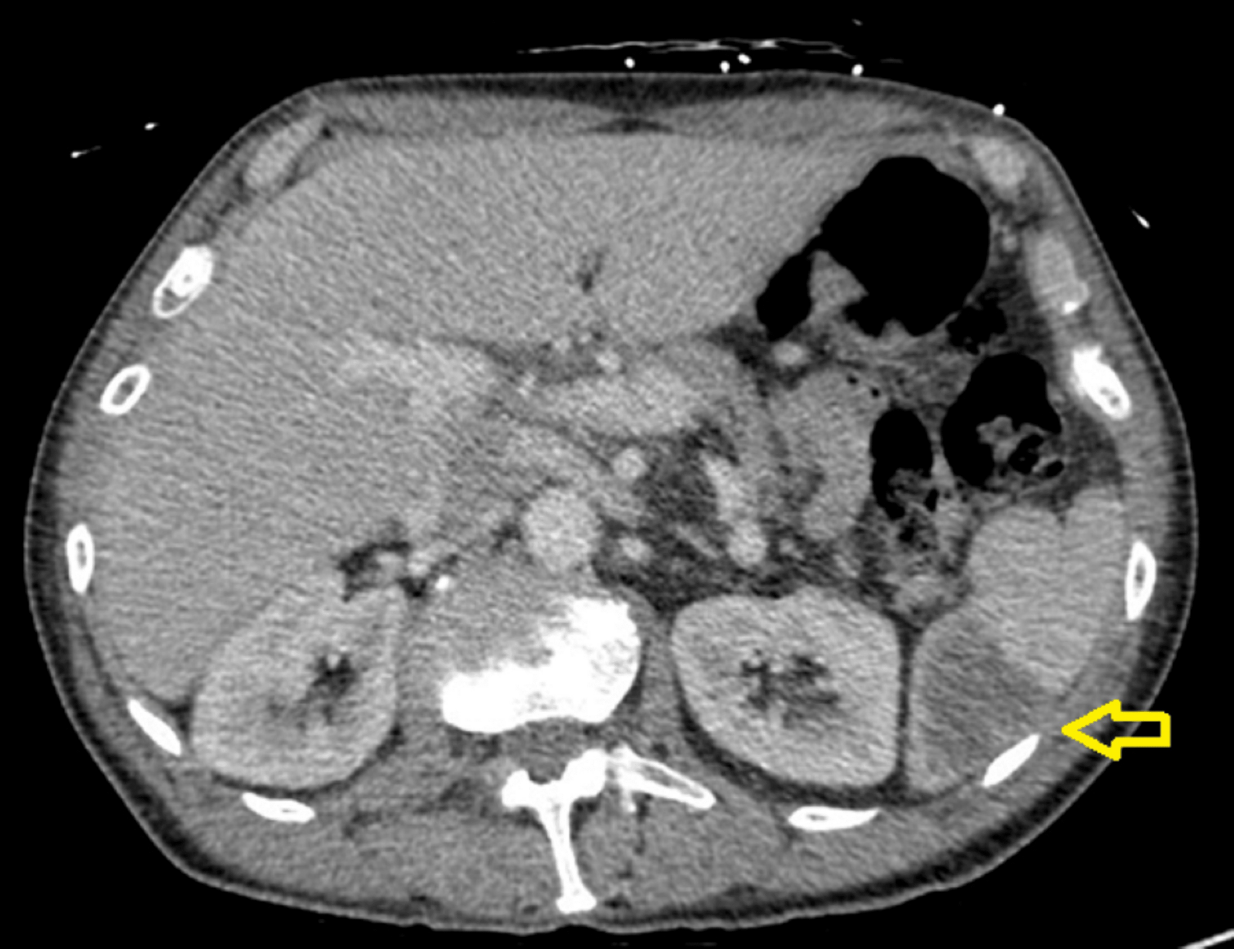
Computed tomography of the abdomen. Wedge-shaped low attenuation along the spleen, likely representing an infarct secondary to septic emboli from endocarditis, as indicated by the yellow arrow.

Cardiothoracic surgery was consulted and recommended surgical repair to include a redo of the mitral valve surgery. Repeat blood cultures did come back positive for *C. hominis*, likely from infection of the prosthetic mitral valve. This was a HACEK PVE, most commonly susceptible to third-generation cephalosporins. Guidelines recommend six weeks of intravenous ceftriaxone for HACEK PVE if the organism is sensitive. Sensitivities for this organism were limited due to the difficulty of growing this fastidious bacterium in a laboratory setting. Infectious disease specialists determined it was best to have the patient continue on intravenous ceftriaxone for six weeks. While admitted, the patient was diagnosed with bioprosthetic mitral valve endocarditis and *C. hominis* bacteremia. Additionally, the patient’s hospitalization was further complicated by a significant decrease in hemoglobin and elevated lactate dehydrogenase levels; it was later determined that his anemia was likely explained by macroangiopathic hemolysis due to a paravalvular leak (Table 1). The patient was also found to have septic emboli to the spleen, with splenic infarcts found incidentally on CT of the abdomen and pelvis (Figure 2). The patient was diagnosed with bioprosthetic mitral valve endocarditis and *C. hominis* bacteremia and was discharged home on six weeks of parenteral ceftriaxone. At a follow-up appointment after six weeks, the patient had complete resolution of symptoms.

**Table 1:** Laboratory evaluation. Relevant laboratory values.

Time	One year before admission	At admission	10 days into admission	Discharge
Hemoglobin level (Reference range: 13.5-17.5 g/dL)	15.4	13.6	10.8	9.1
Lactate dehydrogenase (Reference range: 135-225 U/L)			280	

## Discussion

PVE is a rare but serious condition associated with high mortality and morbidity. This is especially true when caused by unusual pathogens such as *C. hominis*. In this case, we present a patient who had a recent dental procedure without appropriate antibiotic prophylaxis, which is a well-established risk factor for the development of infective endocarditis in patients with prosthetic valves or repaired native cardiac valves [8,9]. This case is notable due to the rarity of *C. hominis* as the causative agent, with only 61 reported cases of this pathogen causing infective endocarditis, and only 15 cases involving PVE.

The rarity of this pathogen is likely related to the unique fastidious nature of *C. hominis* and other HACEK organisms. These organisms are notoriously difficult to grow in standard lab media, making them difficult to identify. This aspect also leads to delayed sensitivities for proper identification and treatment, often resulting in further disease progression and complications [10]. As we observed in this patient who developed macroangiopathic hemolysis due to a paravalvular leak and splenic infarct from undiagnosed PVE, the delay in diagnosis can result in significantly higher mortality and morbidity [11].

The pathogenesis of *C. hominis* endocarditis is not fully understood, but it is thought to be facilitated by the organism's ability to adhere to damaged heart valves, especially those that are prosthetic [12]. The patient in this case had a bioprosthetic valve, which may have been more susceptible to infection following his recent dental procedures. In addition, *C. hominis* has been associated with the formation of large vegetations on heart valves and septic emboli, as evidenced by the TEE findings in this patient and subsequent splenic infarct [13].

Dental procedures are a well-known risk factor for infective endocarditis, especially in patients with prosthetic valves [8,9]. The American Heart Association (AHA) guidelines recommend prophylactic antibiotics for patients with prosthetic valves undergoing invasive dental procedures to prevent the development of PVE [14]. The most commonly recommended antibiotic for this purpose is amoxicillin. However, recent studies have shown that two isolated strains of *C. hominis* produce beta-lactamase, which can render amoxicillin ineffective [15]. This highlights the importance of performing susceptibility testing for patients who develop endocarditis caused by *C. hominis*. In this case, the patient was treated empirically with ceftriaxone, a third-generation cephalosporin, due to the limited sensitivity data available from the obtained blood culture.

Given the rarity of *C. hominis* as a causative agent of PVE, early diagnosis and treatment are vital for improving patient outcomes. While *C. hominis* endocarditis is rare, it is important for clinicians to keep these unusual pathogens in the differential diagnosis, particularly in patients with prosthetic heart valves who are at an increased risk for infective endocarditis from recent procedures. In such cases, timely identification of the organism and appropriate antibiotic therapy are essential to prevent the progression of the disease and mortality and morbidity.

## Conclusions

This case highlights the unique diagnostic and therapeutic challenges that come with infective endocarditis caused by rare pathogens like *C. hominis*. *C. hominis* infective endocarditis is exceedingly rare; however, it can cause significant morbidity and mortality, making it vital to discuss. If not diagnosed and treated appropriately, patients can develop severe sequelae, leading to loss of life and impairment in quality of life. Clinicians should include *C. hominis* in the differential diagnosis of infective endocarditis, particularly in patients with prosthetic heart valves and a history of invasive procedures. Early diagnosis, tailored antibiotic therapy, and adherence to prophylaxis guidelines are essential for improving outcomes in these rare and challenging cases.
